# The Modern Treatment of Epilepsy

**Published:** 1901-01

**Authors:** 


### THE MODERN TREATMENT OF EPILEPSY.

IT HAS been justly said that there is nothing new under the sun, and the review of the modern treatment of epilepsy might emphasize the truth of the saying. Notwithstanding this fact, it must be admitted that a rearrangement of the principles of treatment and their close application to the individual case, is a marked advance over the former desultory drug prescribing. The demands today are that the physician should watch and regulate the vegetative hygienic and psychic life. All disordered bodily functions of the epileptic must be corrected; while the daily activity of the individual patient, his work, education, and play, must be used reflexly to counteract the baleful influence of an inborn convulsive predisposition. In brief, the whole life of the epileptic must be reëstablished on broad and comprehensive principles to aid and round out the drug treatment of the disease.

We find bromides still ranking in the first place as the most efficacious drugs. Clark, of the Craig Colony for Epileptics, believes, after an extensive experience with the drug in the most pronounced cases, that bromides must be given by carefully graduated doses, in amounts ranging from 300 to 400 grains daily, or until the seizures are stopped. He maintains, nevertheless, that extreme caution in giving the drug must be exercised; collateral tonics, massage, and baths, must be unremittingly employed to counteract the general sedative effect on mind and body.

In the surgical treatment, conservatism seems to be the order of the day. Pure idiopathic epileptics should not be trephined for their disease; even a definite order of muscular involvement should not cause us to operate in any idiopathic case. The earlier the prognosis for operation in traumatics the better. We should be very guarded as to recovery or even improvement in traumatic cases in which neuropathic histories are prominent. It should be borne in mind that cases not infrequently are made worse by trephining; it therefore should not be lightly recommended even in traumatic epileptics; statistics show about 4 per cent. of recovery by operating, which result is as good as medical treatment in idiopathics. Post-operative treatment by bromides must be instituted and followed faithfully for several years. We venture to say that by closer diagnosis and treatment, both surgically and medically, the percentage of improvement and recoveries will be increased materially in the future.

